# The efficacy and safety of interscalene blockade versus local infiltration analgesia in primary total shoulder arthroplasty? A protocol for systematic review and meta-analysis of randomized controlled trials

**DOI:** 10.1097/MD.0000000000025201

**Published:** 2021-03-26

**Authors:** Yanhui Wu, Yuan Chen, Cheng Ji, Wen Ye

**Affiliations:** a Department of Anesthesiology; b Department of Orthopedics, Affiliated Hangzhou First People's Hospital, Zhejiang University School of Medicine, Zhejiang, 310006, China.

**Keywords:** interscalene blockade, local infiltration analgesia, meta-analysis, pain control, protocol, total shoulder arthroplasty

## Abstract

**Background:**

None of review has been conducted to compare the efficacy of interscalene blockade (ISB) with that of local infiltration analgesia (LIA) in patients undergoing total shoulder arthroplasty (TSA). We thus conduct a high-quality meta-analysis of randomized controlled trials (RCTs) to investigate which analgesic provides better pain relief.

**Methods:**

A comprehensive search of the published literature in PUBMED, Scopus, EMBASE, and Cochrane Library databases will be performed. Only RCTs evaluating LIA versus ISB in TSA are included in this study. The primary outcome was pain score. Secondary outcome measures included opioid consumption, postoperative adverse event, and length of stay. The Cochrane risk of bias tool is used to evaluate the risk of bias of included RCTs by 2 independent reviewers.

**Results:**

The results of this research will be delivered in a peer-reviewed journal.

**Conclusions:**

This study expects to provide credible and scientific evidence for the efficacy and safety of ISB and LIA for early postoperative pain control after TSA.

**Systematic review registration number:**

10.17605/OSF.IO/S3MBP.

**Ethical approval:**

Since this study is on the basis of published or registered RCTs, ethical approval and informed consent of patients are not required.

## 1 Introduction

Currently, total shoulder arthroplasty (TSA) has been widely used in treatment for patients with degenerative arthritis and rotator-cuff-deficient conditions of the glenohumeral joint.^[1,2]^ The annual number of TSA is rising with the growing elderly population. The growth rates of TSA are higher than the rates for total hip and knee procedures in the United States and were predicted to further increase by between 192% and 322% by 2015 based on 2008 numbers.^[3]^ However, due to the soft tissue injury and large amount of bone destruction involved, undesirable postoperative pain remains a challenge for both patients and surgeons after TSA.

Pain management after TSA is an important variable in the perioperative period that can influence participation in physical therapy, discharge from the hospital or outpatient surgery center, and patient satisfaction. In addition, inadequate pain management has been shown to contribute to an increased incidence of postoperative complications.^[4]^ In shoulder literature, various techniques can be used to relieve postoperative pain, including anti-inflammatory medications, opioids, epidural anesthesia, interscalene blockade (ISB), and local infiltration analgesia (LIA). The ISB allows delivery of local anesthetic in a controlled manner to the trunks of the brachial plexus between the anterior and middle scalene muscles.^[5]^ Compared with patients receiving only general anesthesia, patients who undergo preoperative ISB have shorter hospital stays and a reduced need for analgesics.^[6]^ However, ISB is also associated with risks, such as failure of nerve blockade, residual neurapraxia, displacement of the catheter postoperatively, systemic toxicity, and respiratory and neurologic complications.^[7]^ LIA has recently gained popularity for its potential to provide extended postoperative pain relief. It is a surgeon-controlled analgesic technique that used to reduce the pain in the early postoperative period with no influence on muscle strength. However, the possibility of LIA replacing ISB as an integral component of a multimodal clinical pathway for TSA needs to be further investigated.

However, limited review has been conducted to compare the efficacy of ISB with that of LIA in patients undergoing TSA. Since several randomized controlled trials (RCTs) have been recently published, we thus conduct a high-quality meta-analysis to investigate which analgesic provides better pain relief.^[8–10]^

## 2 Materials and methods

### 2.1 Study registration

The systematic review protocol has been registered on Open Science Framework (OSF) registries. The registration number is 10.17605/OSF.IO/S3MBP. The systematic literature review is structured to adhere to PRISMA guidelines (Preferred Reporting Items for Systematic Reviews and Meta-analyses), which include requirements deemed essential for the transparent reporting of results. We will update our protocol for any changes in the entire research process if needed.^[11]^

### 2.2 Data sources and search strategy

**Figure 1:**
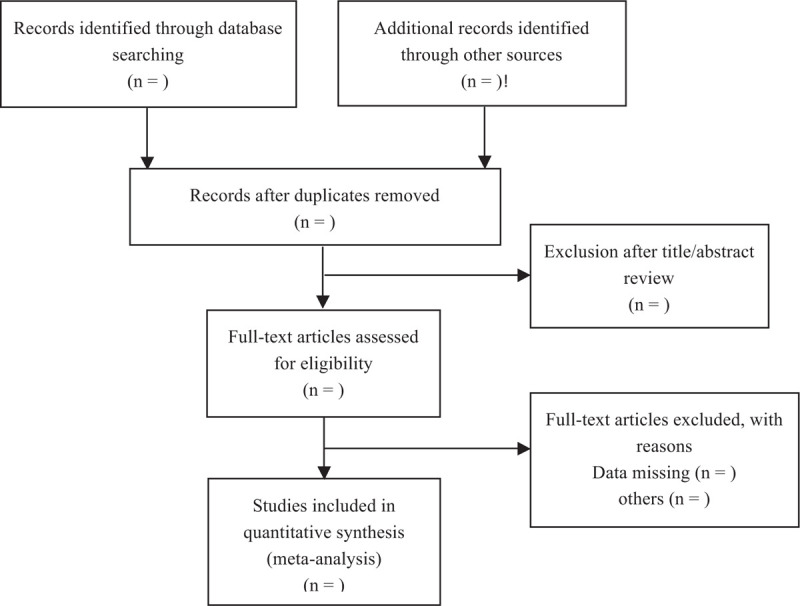
PRISMA flow diagram describing the selection process for relevant clinical trials used in this meta-analysis.

The following search terms will be used in PUBMED, Scopus, EMBASE, and Cochrane Library databases on January 24, 2021, as the search algorithm: (total shoulder arthroplasty) OR (total shoulder replacement) AND (local infiltration analgesia) OR (liposomal bupivacaine) AND (interscalene blockade) OR (interscalene catheter). Two searchers will independently draft and carry out the search strategy, and the third member will further complete it. No time limit is given to publication date. References within included articles are reviewed to include articles that are not included within our literature search. A flow diagram explaining the literature search strategy and study selection is shown in Figure 1.

### 2.3 Eligibility criteria

Studies included in our meta-analysis have to meet all of the following inclusion criteria in the PICOS order:

(1) population: patients undergoing primary TSA;
(2) intervention: ISB group;
(3) comparison intervention: LIA group;
(4) outcome measures: at least one of the following outcome measures is reported: postoperative pain relief, opioid consumption, functional outcome, length of stay, and postoperative adverse event;
(5) study design: English RCTs. Articles with no assessment of outcomes mentioned above or no comparison of 2 groups will not be included into meta-analysis.

Duplicate reports and conference abstracts will be excluded. Retrospective trials, case reports, biochemical trials, letters, and reviews will also be eliminated. Articles are exported to EndNote, and duplicates removed. Two independent authors (YC and JP) screen the titles and abstracts of potentially relevant studies to determine their eligibility based on the criteria.

### 2.4 Data extraction

The method of data extraction will follow the approach outlined by the *Cochrane Handbook for Systematic Reviews of Interventions*.^[12]^ Two independent authors (YC And JP) extract the following descriptive raw information from the selected studies: study characteristics such as author, publication year, study design; patient demographic details such as patients’ number, average age, body mass index, and gender ratio. The primary outcome is pain score. Secondary outcome measures include opioid consumption, postoperative adverse event, and length of stay. Where disagreement in the collection of data occurs, this is resolved through discussion. If the data are missing or cannot be extracted directly, we will contact the corresponding authors to ensure that the information integrated. Otherwise, we calculate them with the guideline of *Cochrane Handbook for Systematic Reviews of Interventions*.^[12]^ If necessary, we will abandon the extraction of incomplete data. A detailed description of all included studies will be listed in Table 1.

**Table 1:** Characteristics of the included studies.

Measured variable	Study 1	Study 2	Study 3	Study 4	Study 5	Study 6	Study 7	Weighted average
Number of patients in ISB								
Number of patients in LIA								
Mean age of ISB								
Mean age of LIA								
Female of ISB								
Female of LIA								
Mean BMI of ISB								
Mean BMI of LIA								
Postop pain score on POD 0 in ISB								
Postop pain score on POD 0 in LIA								
Postop pain score on POD 1 in ISB								
Postop pain score on POD 1 in LIA								
Postop pain score on POD 2 in ISB								
Postop pain score on POD 2 in LIA								
Cumulative opioid consumption on POD 0 in ISB								
Cumulative opioid consumption on POD 0 in LIA								
Cumulative opioid consumption on POD 1 in ISB								
Cumulative opioid consumption on POD 1 in LIA								
Cumulative opioid consumption on POD 2 in ISB								
Cumulative opioid consumption on POD 2 in LIA								
Mean length of stay in ISB								
Mean length of stay in LIA								

BMI = body mass index, ISB = interscalene blockade, LIA = local infiltration analgesia, NR = not reported, POD = postoperative day.

### 2.5 Statistical analysis

Review Manager software (v 5.3; Cochrane Collaboration) is used for the meta-analysis. Extracted data are entered into Review Manager by the first independent author and checked by the second independent author. Risk ratio with a 95% confidence interval or standardized mean difference with 95% CI are assessed for dichotomous outcomes or continuous outcomes, respectively. The heterogeneity is assessed by using the *Q* test and *I*^2^ statistic. An *I*^2^ value of <25% is chosen to represent low heterogeneity and an *I*^2^ value of >75% to indicate high heterogeneity. All outcomes are pooled on random-effect model. A *P* value of <.05 is considered to be statistically significant.

### 2.6 Quality assessment

The Cochrane risk of bias tool^[12]^ is used to evaluate the risk of bias of included RCTs by 2 independent reviewers. The quality of RCTs is assessed by using following 7 items: random sequence generation, allocation concealment, blinding of participants and personnel, blinding of outcome assessment, incomplete outcome data, selective reporting, and other bias (Table 2). Disagreement is resolved through discussion and consensus between the reviewers. Kappa values will be used to measure the degree of agreement between the 2 reviewers and are rated as follows: fair, 0.40 to 0.59; good, 0.60 to 0.74; and excellent, 0.75 or more. Based on the information provided from included studies, each item is recorded as low risk of bias, high risk of bias, or unclear (lack of information or unknown risk of bias). We also conduct the sensitivity analysis to evaluate whether any single study have the weight to skew on the overall estimate and data. Begg's funnel plot is used to assess publication bias. If publication bias exists, the Begg's funnel plot is asymmetric.

**Table 2:** Methodological assessment according to 6 domains of potential biases (Cochrane risk of bias tool).

RCT study = 7	Sequence generation	Allocation concealment	Blinding of participants and personnel	Blinding of outcome assessors	Incomplete outcome data	Selective outcome reporting	Other potential threats to validity	Overall bias
Study 1								
Study 2								
Study 3								
Study 4								
Study 5								
Study 6								
Study 7								

## 3 Discussion

In recent years, the use of TSA for treatment of various shoulder conditions has grown rapidly. Considering the continuously increasing number of TSA being performed, establishing a reasonable postoperative pain control program has become essential. The aim of the present study was to compare the efficacy of ISB and LIA in postoperative pain control after TSA.

Several studies have compared the efficacy of ISB vs LIA for pain management following TSA. Angerame et al found no significant differences in mean pain level, postoperative opioid consumption, or length of stay, indicating that LIA may be as effective as ISB therapy.^[13]^ Hannan et al conducted a cohort study and revealed that LIA was associated with less pain, less opioid consumption, and shorter hospital stays after TSA compared with ISB.^[14]^ The study by Weller et al showed that LIA was associated with similar pain relief, increased opioid consumption, and fewer complications compared to ISB following TSA.^[15]^ However, these studies were retrospective cohort studies. With reduced opioid consumption, patients in ISB group can mitigate potential adverse events such as respiratory depression, nausea, and vomiting. Perhaps increased levels of narcotics consumed in the LIA cohort could very well offset any potential advantages over the potential complications associated with ISB.

Numerous meta-analyses have been published comparing the pain management of ISB and LIA in TSA. In a meta-analysis that included 2 RCTs^[6,16]^ and 2 retrospective studies,^[14,15]^ Yan et al indicated that compared with ISB, LIA with liposomal bupivacaine had comparative effectiveness on reducing both pain scores and length of hospital stay.^[17]^ Wang et al^[18]^ including 4 RCTs^[4,6,15,16]^ reported similar effectiveness of pain relief between ISB and LIA. However, the author mistakenly used a retrospective study^[15]^ as RCT in their study. In the study of Sun et al,^[19]^ the authors included 4 RCTs^[4,6,16,20]^ and 3 retrospective studies^[13–15]^ to compare ISB alone with LIA alone and found that both techniques provide similar overall pain relief and have similar opioid consumption, while patients treated with LIA experienced significantly less occurrence rate of complications after TSA. However, they contained some methodological shortcomings, data extraction, limited sample size, and high heterogeneity. Since several RCTs have been recently published, we thus conduct an updated high-quality meta-analysis to investigate which analgesic provides better pain relief. The results of this research will be delivered in a peer-reviewed journal. This study expects to provide credible and scientific evidence for the efficacy and safety of ISB and LIA for early postoperative pain control after TSA.

## Author contributions

**Conceptualization:** Cheng Ji.

**Data curation:** Yanhui Wu, Yuan Chen.

**Formal analysis:** Yanhui Wu.

**Funding acquisition:** Wen Ye.

**Investigation:** Yanhui Wu, Yuan Chen.

**Methodology:** Yuan Chen, Cheng Ji.

**Project administration:** Wen Ye.

**Software:** Cheng Ji.

**Supervision:** Wen Ye.

**Validation:** Cheng Ji.

**Visualization:** Yuan Chen.

**Writing – original draft:** Yanhui Wu.

**Writing – review & editing:** Wen Ye.
